# Adjuvant chemoradiotherapy versus radiotherapy alone for women with high-risk endometrial cancer (PORTEC-3): final results of an international, open-label, multicentre, randomised, phase 3 trial

**DOI:** 10.1016/S1470-2045(18)30079-2

**Published:** 2018-03

**Authors:** Stephanie M de Boer, Melanie E Powell, Linda Mileshkin, Dionyssios Katsaros, Paul Bessette, Christine Haie-Meder, Petronella B Ottevanger, Jonathan A Ledermann, Pearly Khaw, Alessandro Colombo, Anthony Fyles, Marie-Helene Baron, Ina M Jürgenliemk-Schulz, Henry C Kitchener, Hans W Nijman, Godfrey Wilson, Susan Brooks, Silvestro Carinelli, Diane Provencher, Chantal Hanzen, Ludy C H W Lutgens, Vincent T H B M Smit, Naveena Singh, Viet Do, Romerai D'Amico, Remi A Nout, Amanda Feeney, Karen W Verhoeven-Adema, Hein Putter, Carien L Creutzberg, PORTEC study group, Mary McCormack, Karen Whitmarsh, Rozenn Allerton, Deborah Gregory, Paul Symonds, Peter J. Hoskin, Madhavi Adusumalli, Anjana Anand, Robert Wade, Alexandra Stewart, Wendy Taylor, Roy F.P.M. Kruitwagen, Harry Hollema, Elizabeth Pras, An Snyers, Lukas Stalpers, Jan J. Jobsen, Annerie Slot, Jan-Willem M. Mens, Tanja C. Stam, Baukelien Van Triest, Elzbieta M. Van der Steen - Banasik, Karin A.J. De Winter, Michael A. Quinn, Ilka Kolodziej, Jan Pyman, Carol Johnson, Anne Capp, Roldano Fossati, Sergio Gribaudo, Andrea A. Lissoni, Annamaria Ferrero, Grazia Artioli, Cathy Davidson, C. Meg McLachlin, Prafull Ghatage, Paula V.C. Rittenberg, Luis Souhami, Gillian Thomas, Pierre Duvillard, Dominique Berton-Rigaud, Nicole Tubiana-Mathieu

**Affiliations:** a Department of Radiation Oncology, Leiden University Medical Center, Leiden, Netherlands; b Department of Pathology, Leiden University Medical Center, Leiden, Netherlands; c Department of Medical Statistics, Leiden University Medical Center, Leiden, Netherlands; d Department of Clinical Oncology, Barts Health NHS Trust, London, UK; e Department of Cellular Pathology, Barts Health NHS Trust, London, UK; f Division of Cancer Medicine, Peter MacCallum Cancer Centre, Melbourne, VIC, Australia; g Division of Radiation Oncology, Peter MacCallum Cancer Centre, Melbourne, VIC, Australia; h Department of Surgical Sciences, Gynecologic Oncology, Città della Salute and S Anna Hospital, University of Turin, Turin, Italy; i CCTG, Department of Obstetrics and Gynaecology, University of Sherbrooke, Sherbrooke, QC, Canada; j Department of Radiotherapy, Institut Gustave Roussy, Villejuif, France; k Department of Medical Oncology, Radboudumc, Nijmegen, Netherlands; l Cancer Research UK, London, UK; m UCL Cancer Trials Centre, UCL Cancer Institute, London, UK; n Division of Radiation Oncology, ASST-Lecco, Ospedale AManzoni, Lecco, Italy; o CCTG, Radiation Medicine Program, Princess Margaret Cancer Centre, Toronto, ON, Canada; p Department of Radiotherapy, Centre Hospitalier Régional Universitaire de Besançon, Besançon, France; q Department of Radiation Oncology, University Medical Center Utrecht, Netherlands; r Institute of Cancer Sciences, University of Manchester, Manchester, UK; s Department of Gynaecologic Oncology, University Medical Center Groningen, University of Groningen, Groningen, Netherlands; t Department of Pathology, Central Manchester Hospitals NHS Foundation Trust, Manchester Royal Infirmary, Manchester, UK; u Department of Radiation Oncology, Auckland City Hospital, Auckland, New Zealand; v Division of Pathology and Laboratory Medicine, European Institute of Pathology, Milan, Italy; w Department of Gynaecologic Oncology, HÔpital Notre-Dame de Montreal, Montreal, QC, Canada; x Department of Radiation Oncology, Centre Henri Becquerel, Rouen, France; y Department of Radiation Oncology (MAASTRO), GROW School for Oncology and Developmental Biology, Maastricht University Medical Centre, Maastricht, Netherlands; z Radiation Oncology Network, CPMCC Westmead, Westmead, NSW, Australia; aa Comprehensive Cancer Center Netherlands, Leiden, Netherlands

## Abstract

**Background:**

Although women with endometrial cancer generally have a favourable prognosis, those with high-risk disease features are at increased risk of recurrence. The PORTEC-3 trial was initiated to investigate the benefit of adjuvant chemotherapy during and after radiotherapy (chemoradiotherapy) versus pelvic radiotherapy alone for women with high-risk endometrial cancer.

**Methods:**

PORTEC-3 was an open-label, international, randomised, phase 3 trial involving 103 centres in six clinical trials collaborating in the Gynaecological Cancer Intergroup. Eligible women had high-risk endometrial cancer with FIGO 2009 stage I, endometrioid-type grade 3 with deep myometrial invasion or lymph-vascular space invasion (or both), endometrioid-type stage II or III, or stage I to III with serous or clear cell histology. Women were randomly assigned (1:1) to receive radiotherapy alone (48·6 Gy in 1·8 Gy fractions given on 5 days per week) or radiotherapy and chemotherapy (consisting of two cycles of cisplatin 50 mg/m^2^ given during radiotherapy, followed by four cycles of carboplatin AUC5 and paclitaxel 175 mg/m^2^) using a biased-coin minimisation procedure with stratification for participating centre, lymphadenectomy, stage of cancer, and histological type. The co-primary endpoints were overall survival and failure-free survival. We used the Kaplan-Meier method, log-rank test, and Cox regression analysis for final analysis by intention to treat and adjusted for stratification factors. The study was closed on Dec 20, 2013, after achieving complete accrual; follow-up is ongoing. PORTEC-3 is registered with ISRCTN, number ISRCTN14387080, and ClinicalTrials.gov, number NCT00411138.

**Results:**

686 women were enrolled between Nov 23, 2006, and Dec 20, 2013. 660 eligible patients were included in the final analysis, of whom 330 were assigned to chemoradiotherapy and 330 were assigned to radiotherapy. Median follow-up was 60·2 months (IQR 48·1–73·1). 5-year overall survival was 81·8% (95% CI 77·5–86·2) with chemoradiotherapy versus 76·7% (72·1–81·6) with radiotherapy (adjusted hazard ratio [HR] 0·76, 95% CI 0·54–1·06; p=0·11); 5-year failure-free survival was 75·5% (95% CI 70·3–79·9) versus 68·6% (63·1–73·4; HR 0·71, 95% CI 0·53–0·95; p=0·022). Grade 3 or worse adverse events during treatment occurred in 198 (60%) of 330 who received chemoradiotherapy versus 41 (12%) of 330 patients who received radiotherapy (p<0·0001). Neuropathy (grade 2 or worse) persisted significantly more often after chemoradiotherapy than after radiotherapy (20 [8%] women *vs* one [1%] at 3 years; p<0·0001). Most deaths were due to endometrial cancer; in four patients (two in each group), the cause of death was uncertain. One death in the radiotherapy group was due to either disease progression or late treatment complications; three deaths (two in the chemoradiotherapy group and one in the radiotherapy group) were due to either intercurrent disease or late treatment-related toxicity.

**Interpretation:**

Adjuvant chemotherapy given during and after radiotherapy for high-risk endometrial cancer did not improve 5-year overall survival, although it did increase failure-free survival. Women with high-risk endometrial cancer should be individually counselled about this combined treatment. Continued follow-up is needed to evaluate long-term survival.

**Funding:**

Dutch Cancer Society, Cancer Research UK, National Health and Medical Research Council Project Grant and Cancer Australia, L'Agenzia Italiana del Farmaco, and Canadian Cancer Society Research Institute.

**Research in context**

**Evidence before this study**

We searched PubMed for clinical studies published in English between Jan 1, 1980, and Dec 31, 2006, with the terms “endometrial cancer” AND “radiation therapy” AND “chemotherapy” AND “survival” OR “failure free survival”. We identified six relevant publications. Three randomised controlled trials compared chemotherapy with radiotherapy. The GOG-122 trial compared whole abdominal radiotherapy with doxorubicin–cisplatin chemotherapy in patients with stage III or IV endometrial cancer. In adjusted analysis, improved progression-free survival and overall survival were reported for patients treated with chemotherapy, but with high proportions of patients with toxicity and similar event rates in both groups. Two trials (one in Italy and one in Japan) compared pelvic radiotherapy with three cycles or five cycles of cyclophosphamide–doxorubicin–cisplatin chemotherapy in stage I–III disease and neither showed any difference in overall survival or relapse-free survival. In the Italian trial, most patients had stage III disease, and chemotherapy delayed distant metastases and radiotherapy delayed pelvic recurrence, but without differences in overall survival or progression-free survival. Because of increased pelvic relapse with the use of adjuvant chemotherapy alone, the combination of chemotherapy and radiotherapy merited exploration. The phase 2 RTOG-9708 trial, which assessed toxicity on the chemoradiotherapy schedule on which PORTEC-3 is based, reported 4-year overall survival of 85% and disease-free survival of 81%. Since the start of recruitment to the PORTEC-3 trial, the results of three randomised trials comparing chemoradiotherapy with radiotherapy alone have been published. A small Finnish trial compared radiotherapy alone with chemotherapy plus radiotherapy given in two courses of 28 Gy each; no difference in overall survival or recurrence was reported. The NSGO-EC-9501/EORTC-55991 trial was published in a pooled analysis with the unfinished ManGO Iliade phase 3 trial, and showed significantly improved progression-free survival and a trend for improved overall survival with the addition of four cycles of platinum-based chemotherapy given sequentially before or after pelvic radiotherapy. Two more trials (GOG-249 and GOG-258) have not yet been fully published, but have been presented as abstracts at conferences. The results of the GOG-249 trial, which compared pelvic radiotherapy with a combination of three cycles of carboplatin-paclitaxel chemotherapy and vaginal brachytherapy in stage I–II patients with high (intermediate) risk factors reported overlapping progression-free survival and overall survival curves, and significantly more pelvic and para-aortic recurrences in the chemotherapy group. The GOG-258 trial compared chemoradiotherapy (the same schedule as used in the PORTEC-3 trial) with six cycles of carboplatin–paclitaxel chemotherapy alone. No differences in overall or recurrence-free survival were reported, but significantly more vaginal and pelvic or para-aortic recurrences were reported in the chemotherapy group.

**Added value of this study**

We report the overall survival and failure-free survival of patients with high-risk endometrial cancer treated in the international PORTEC-3 trial. Patients were randomly assigned to receive pelvic radiotherapy alone or radiotherapy combined with concurrent (two cycles of cisplatin) and adjuvant (four cycles of carboplatin–paclitaxel) chemotherapy. The addition of chemotherapy to adjuvant radiotherapy significantly improved failure-free survival compared with radiotherapy alone, but not overall survival. Vaginal and pelvic control was high with radiotherapy in both groups. The treatment duration was longer in the chemoradiotherapy group than in the radiotherapy group, and significantly higher rates of adverse events were reported in the chemoradiotherapy group during, and in the first year after, treatment.

**Implications of all the available evidence**

Combined adjuvant chemotherapy and radiotherapy cannot be recommended as a new standard of care for patients with stage I–II endometrial cancer because no survival differences were found and pelvic control was high with radiotherapy alone. Patients with stage III cancer had the greatest benefit with chemoradiotherapy because of their higher risk of disease recurrence; for these patients, combined treatment should be considered to maximise failure-free survival. Nevertheless, the benefits and risks should be discussed for each individual patient.

## Introduction

The majority of women with endometrial cancer present with early-stage disease and have a favourable prognosis. About 15% of women with endometrial cancer are diagnosed with high-risk disease, which comprises endometrioid endometrial cancer stage I, grade 3 with deep invasion or with lymph-vascular space invasion (LVSI), stage II or III endometrioid endometrial cancer, or non-endometrioid (serous or clear cell) histology. Women with high-risk endometrial cancer are at increased risk of distant metastases and cancer-related death.^1^, ^2^, ^3^, ^4^ Serous and clear cell cancers have a higher risk of aggressive spread and a worse prognosis; however, in the early stages they have similar outcomes to grade 3 endometrioid endometrial cancer.^5^

Pelvic external beam radiotherapy has been the standard adjuvant treatment for women with high-risk endometrial cancer for many decades, although there is a paucity of evidence on improvement of survival. Randomised trials^6^, ^7^ have compared adjuvant chemotherapy with external beam radiotherapy. Radiotherapy was shown to delay pelvic recurrence and chemotherapy was shown to delay distant metastases, but no differences in survival were found.

Because increased incidence of pelvic relapse has been reported with chemotherapy alone, the combination of external beam radiotherapy with chemotherapy has been explored. In a phase 2 trial (RTOG 9708)^8^ among women with high-risk endometrial cancer, the combination of external beam radiotherapy with two concurrent cycles of cisplatin, followed by four adjuvant cycles of cisplatin and paclitaxel, was tested, resulting in 4-year overall survival of 85% and disease-free survival of 81%.

Because the combination of radiotherapy and chemotherapy (chemoradiotherapy) seemed more effective than either treatment alone, and because data for toxicity and quality of life were lacking, the randomised PORTEC-3 trial was initiated to evaluate the benefit of chemoradiotherapy versus radiotherapy alone for women with high-risk endometrial cancer in terms of overall survival and failure-free survival improvement, as well as toxicity and effects on health-related quality of life. Analysis of 2-year toxicity and health-related quality of life in the PORTEC-3 trial showed significantly higher rates of adverse events and reduced health-related quality of life during and after chemoradiotherapy treatment, with rapid recovery thereafter.^9^

Here, we present the final analysis of the primary survival endpoints of the PORTEC-3 trial.

## Methods

### Study design and participants

PORTEC-3 was an open-label, randomised, phase 3 trial at 103 centres (oncology centres, university hospitals, regional hospitals, or radiation oncology centres with referrals from regional hospitals) in six clinical trial groups collaborating in the Gynaecological Cancer Intergroup. Participating groups were the National Cancer Research Institute (NCRI; UK), Australia and New Zealand Gynaecologic Oncology Group (ANZGOG; Australia and New Zealand), Mario Negri Gynaecologic Oncology Group (MaNGO; Italy), Canadian Cancer Trials Group (CCTG; Canada), and Fedegyn (France).

Patients were eligible if they had endometrial cancer with either International Federation of Gynecology and Obstetrics (FIGO) 2009 stage 1A endometrioid endometrial cancer grade 3 with documented LVSI; stage IB endometrioid endometrial cancer grade 3; stage II endometrioid endometrial cancer; stage IIIA, IIIB (parametrial invasion), or IIIC endometrioid endometrial cancer; or serous or clear-cell histology endometrial cancer with stages IA (with invasion), IB, II, or III. Eligibility also included WHO performance score 0–2; adequate bone marrow function (white blood cells ≥3·0 × 10^9^/L, platelets ≥100 × 10^9^/L), liver function (bilirubin ≤1·5 × upper normal limit [UNL], aspartate aminotransferase and alanine aminotransferase ≤2·5 × UNL), kidney function (creatinine clearance >60 mL per min calculated according to Cockroft and Gault^10^ or >50 mL per min EDTA clearance), and aged 18 years or older (without an upper age limit, because elderly women might benefit from the study treatment if deemed fit enough to undergo chemotherapy). Exclusion criteria were uterine (carcino)sarcoma; malignancy in the 10 years before diagnosis of endometrial cancer; previous pelvic radiotherapy, hormonal therapy, or chemotherapy; bulky cervical involvement with radical hysterectomy; inflammatory bowel disease; residual macroscopic tumour; impaired renal or cardiac function; grade 2 or worse neuropathy; grade 3 or worse hearing impairment; or congenital hearing disorder.

Surgery comprised total abdominal or laparoscopic hysterectomy with bilateral salpingo-oophorectomy. Lymphadenectomy, whether systemic or sampling, was left to the discretion of participating centres, while lymph node debulking and para-aortic lymph-node sampling were recommended in cases of macroscopic positive pelvic nodes or para-aortic nodes (or both). Lymphadenectomy was not mandated in view of the lack of improvement in overall or progression-free survival in early-stage disease and its associated toxicity, mainly lymph oedema.^11^, ^12^ For high-risk disease, the value of lymphadenectomy to direct adjuvant treatment is debated,^13^ and the international STATEC trial^14^ has been initiated to address this issue. For serous or clear-cell carcinoma, full surgical staging (with omentectomy, peritoneal biopsies, and lymph node sampling) was strongly recommended. Central pathology review by the groups' reference gynaecopathologists was required before randomisation to confirm patients' final suitability for study entry.

Written informed consent was obtained from all patients. The protocol was approved by the Dutch Cancer Society and by the Ethics Committees of all participating groups. The study protocol is available online.

### Randomisation and masking

Patients were randomly allocated (1:1) to chemoradiotherapy or radiotherapy alone. Treatment was allocated with a biased-coin minimisation procedure, ensuring balance overall and within each stratum of the stratification factors (participating centre, lymphadenectomy, stage of cancer, and histological type). Patients were registered and randomised by the participating group's data centres and treatment was assigned via a web-based application. The assigned treatment was generated immediately by the randomisation programme and confirmed by email. Participants and investigators were not masked to treatment allocation.

### Procedures

Central pathology review was done by reference gynaecopathologists (as appointed by each participating group before the start of the trial) to determine final eligibility. The slides and blocks were sent to each participating group's central review pathologists at one gynaecological pathology review site (in France and Italy), two sites (in the UK and the Netherlands), or five to six sites (in Australia and New Zealand, and Canada), with the result of the review confirming the patient's eligibility for the trial being sent to the local investigators within 1 week. Details of pathology review and inter-observer variation compared with local pathology assessment are reported separately.^15^ In this analysis, review pathology assessment was used. If any particular details were missing, the original pathology was used for these specific items. LVSI was recorded as present or absent. Extensive LVSI in the parametrial tissues was considered stage IIIB. In case of serosal breach, metastases in the stroma of the fallopian tubes, in the ovaries, or on the peritoneal surface of the tubes or ovaries (or both), the stage was defined as IIIA. After determination of eligibility and patient consent, a tumour sample was centrally stored for future translational research.

External beam pelvic radiotherapy was given in both treatment groups to a total dose of 48·6 Gy in 1·8 Gy fractions, 5 days a week. For 11 of the 32 UK sites, a dose of 45 Gy (1·8 Gy fractions) was allowed if specified before initiation of the trial. The clinical target volume included the proximal vagina, parametrial tissues, and internal, external, and common iliac lymph node regions up to the L5–S1 level. The clinical target volume was extended to include the aortic bifurcation in case of iliac lymph node involvement; to include the lower peri-aortic region for common iliac node involvement; and to include the higher para-aortic region in case of para-aortic involvement (with a margin of ≥2 cm above the highest involved lymph node). If complete bilateral lymphadenectomy had been done with at least 12 lymph nodes, it was recommended to have the upper clinical target volume border at the iliac bifurcation. In case of cervical involvement (glandular, stromal, or both), a brachytherapy boost was given to the vaginal vault. Brachytherapy dose was equivalent to 14 Gy in 2 Gy fractions (with recommended scheme of 10 Gy high-dose rate [HDR] in fractions of 5 Gy), specified at 5 mm from the vaginal vault surface. Most patients were treated with a four-field technique; use of intensity-modulated radiotherapy was allowed for centres per approval by their group's principal investigator.

Treatment was recommended to start within 4–6 weeks of surgery, but no later than 8 weeks. Overall radiotherapy treatment time was not to exceed 50 days. Radiotherapy quality assurance was not initially part of the trial, because pelvic radiotherapy was standard practice and used in both groups. However, the Trans-Tasman Radiation Oncology Group (TROG) initiated a bench-marking and quality assurance programme for the ANZGOG group,^16^ and in 2012, a protocol amendment allowed a short quality-assurance programme to be activated for all other participating sites, with independent review of a single radiotherapy plan for each site.

Patients in the chemoradiotherapy group received two cycles of intravenous cisplatin 50 mg/m^2^ in the first and fourth week of external beam pelvic radiotherapy, followed by four cycles of intravenous carboplatin AUC5 and paclitaxel 175 mg/m^2^ at 21-day intervals. This schedule was based on the RTOG-9708 trial,^8^ with substitution of cisplatin by carboplatin in the adjuvant phase to reduce toxicity and in view of the use of carboplatin–paclitaxel chemotherapy in metastatic disease.^17^

Adjuvant chemotherapy was to be started within 3 weeks after completion of external beam pelvic radiotherapy, and with a 28-day interval from the second concurrent cycle. Toxicity, however, had to be resolved to better than grade 2 before start of chemotherapy.

In the event of toxicities, cisplatin was postponed for 1 week. If recovery required more than 1 week, or in the case of neuropathy of grade 2 or worse, cisplatin was discontinued. Carboplatin was postponed or stopped in case of severe haematological toxicity. Paclitaxel was postponed for grade 2 neuropathy and stopped if recovery exceeded 1 week or grade 3 neuropathy developed. Carboplatin and paclitaxel were delayed for other grade 3–4 toxicities, and discontinued if no recovery or reduction to grade 1 occurred. Details on chemotherapy stopping rules have been described previously.^9^

At each follow-up, patient history was taken with emphasis on toxicities and symptoms of recurrent disease, and physical and pelvic examination were done. Chest radiography, blood count, and chemistry tests (including Ca-125) were to be obtained annually, up to 5 years. Long-term follow-up (by hospital visit or information from the general practitioner) was required at 7 years and 10 years.

### Outcomes

The coprimary endpoints were overall survival and failure-free survival. Overall survival was defined as time from date of randomisation to date of death from any cause. Failure-free survival (defined as any relapse or death related to endometrial cancer or treatment) was defined as time from randomisation to date of first failure-free survival event. Failure-free survival events were evaluated by the central data manager, the chief investigator, and the associated investigator, who were unaware of treatment allocation. Women who were alive at the time of analysis were censored at the date of their last follow-up. Secondary endpoints were vaginal, pelvic, or distant recurrence; treatment-related toxicity; and health-related quality of life (published elsewhere^9^). Recurrences were analysed according to first site of recurrence. Abdominal recurrences outside the pelvic area (peritoneal carcinomatosis, liver, and para-aortic lymph nodal metastases) were considered distant metastases, with specification of site.

Toxicity was assessed and graded with Common Terminology Criteria for Adverse Events (CTCAE) version 3.0^18^ at baseline (after surgery), at completion of radiotherapy, after each chemotherapy cycle, at 6-month intervals from randomisation until 5 years, and at 7 years and 10 years. Grade 2 or worse adverse events were to be reported, regardless of the association with study treatment. For evaluation of mild (grade 1) toxicities, patient-reported health-related quality-of-life symptoms were used because patient reporting of grade 1 toxicities was considered most reliable.^19^ Serious adverse events had to be reported within 24 h, specifying adverse event grade and whether or not they were associated with study treatment.

### Statistical analysis

The PORTEC-3 trial was powered (80%) to detect a 10% difference in 5-year overall survival (increase from 65% to 75%; hazard ratio [HR] 0·67), with a two-sided α value of 0·05. 198 events were required, with a minimum number of 655 patients. The number of required patients was increased to 670 to ensure 655 eligible and evaluable patients. Power calculation of the coprimary endpoint failure-free survival was based on the same principles as overall survival.

The first prespecified interim analysis was done after 48 overall survival events (a third of the required events) had occurred in September, 2013, only 3 months before reaching complete accrual. In October, 2016, we decided, with permission from the Data Safety Monitoring Board (DSMB), not to do the prespecified second interim analysis at two-thirds of overall survival events, because this would have no consequences for the trial and would reduce α-spending. To maintain an overall α of 0·05, with a nominal α level for the first interim analysis of 0·0002, the final analysis was done with a nominal α of 0·0498. For analysis of the coprimary endpoints, overall survival and failure-free survival with a correlation between the test-statistics of the coprimary endpoints of 0·7859 (based on 136 overall survival events and 186 failure-free survival events), a nominal α of 0·0309 was used for each of the analyses, resulting in an overall α level of 0·0498.^20^

Because deaths in the PORTEC-3 trial were lower than expected at the time of trial design, the required number of overall survival events was not expected to be reached before late 2018. Recurrence was highest in the first 3 years after treatment, with a sharp decline thereafter, and relapse was rare after 5 years. For these reasons, the DSMB approved the final analysis becoming time-based rather than event-based, with final analysis at a median follow-up of 5 years and 42 months additional follow-up after inclusion of the last patient.

We did statistical analyses using SPSS version 23.0 and R version 3.2.1. All analyses were done by intention to treat, excluding patients who immediately withdrew informed consent and ineligible patients. Differences in relapse and survival rates between the groups were tested with log-rank test and Cox-regression analysis. The analysis of the primary endpoints was adjusted for the stratification factors (participating group, lymphadenectomy, stage of cancer, and histological type), as the appropriate method when using a stratified minimisation procedure at randomisation.^21^, ^22^ For adjusted analysis, stratification factors were included as covariates in the Cox model. For analysis of failure-free survival and recurrence, competing-risk methods were used.^23^ For failure-free survival, intercurrent death was used as a competing risk. For the first failure analysis of recurrences, all other recurrences and death were used as competing risks. Predictive factors were assessed using Cox regression with treatment-by-covariate interaction including the stratification factors, as well as LVSI and age. The median follow-up was estimated with the reverse Kaplan Meier method.

This study is registered with ISRCTN, number ISRCTN14387080 and ClinicalTrials.gov, number NCT00411138.

### Role of the funding source

The funding bodies had no role in study design, data collection, data interpretation, data analysis, or writing of this report. The central data manager (KWV), the chief investigator (CLC), the associated investigators (SMdB, RAN), and the trial statistician (HP) had full access to all the data. The decision to submit for publication was made after discussion within the trial management group and with approval of the DSMB. The corresponding author and chief investigator had full access to all the data and the final responsibility to submit for publication.

## Results

**Figure 1:**
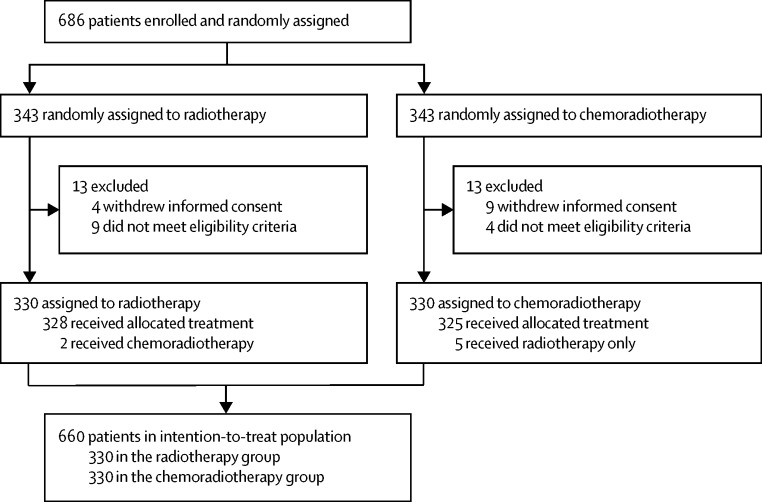
Trial profile

Between Nov 23, 2006, and Dec 20, 2013, 686 women were enrolled and randomly assigned to chemo-radiotherapy (n=343) or radiotherapy (n=343). 26 patients were excluded: 13 because of immediate informed consent withdrawal and 13 because they did not fulfil the eligibility criteria (figure 1). 660 patients were included in the primary analysis (chemoradiotherapy, n=330; radiotherapy, n=330). Median follow-up was 60·2 months (IQR 48·1–73·1) overall and was 60·0 months (47·8–73·1) in the chemoradiotherapy group and 60·7 months (48·7–72·9) in the radiotherapy group. There were seven major protocol violations: in the chemoradiotherapy group, five patients refused chemotherapy and received radiotherapy only; in the radiotherapy group, two patients asked to switch to chemoradiotherapy (figure 1).

Patient characteristics were well balanced between the treatment groups (table 1). The median age was 62 years (IQR 56·2–68·0). Lymphadenectomy, lymph node sampling, or full surgical staging were done in 190 patients (58%) in the chemoradiotherapy group and in 192 patients (58%) in the radiotherapy group.

**Table 1:** Patient, tumour, and treatment characteristics

		**Chemoradiotherapy (n=330)**	**Radiotherapy alone (n=330)**
**Age at randomisation (years)**			
Median		62·4 (56·5–67·9)	62·0 (55·8–68·2)
<60		128 (39%)	140 (42%)
60–69		144 (44%)	128 (39%)
≥70		58 (18%)	62 (19%)
**Participating groups**			
NCRI (UK)		82 (25%)	95 (29%)
DGOG (Netherlands)		72 (22%)	66 (20%)
ANZGOG (Australia and New Zealand)		60 (18%)	58 (18%)
MaNGO (Italy)		52 (16%)	46 (14%)
CCTG (Canada)		36 (11%)	29 (9%)
Fedegyn (France)		28 (9%)	36 (11%)
**FIGO 2009 stage**			
Stage IA		39 (12%)	38 (12%)
Stage IB		59 (18%)	59 (18%)
Stage II		80 (24%)	90 (27%)
Stage III		152 (46%)	143 (43%)
**Histological grade and type**			
EEC grade 1		68 (21%)	56 (17%)
EEC grade 2		59 (18%)	73 (22%)
EEC grade 3		90 (27%)	95 (29%)
Serous		53 (16%)	52 (16%)
Clear cell		29 (9%)	33 (10%)
Mixed		17 (5%)	13 (4%)
Other		14 (4%)	8 (2%)
**Myometrial invasion**			
<50%		116 (35%)	123 (37%)
≥50%		212 (65%)	206 (63%)
Missing		2 (<1%)	1 (<1%)
**LVSI**			
Yes		197 (60%)	192 (58%)
No		133 (40%)	138 (42%)
**WHO performance score**			
0–1		323 (99%)	324 (99%)
≥2		5 (2%)	5 (2%)
Missing		2 (<1%)	1 (<1%)
**Comorbidity**			
Diabetes		45 (14%)	36 (11%)
Hypertension		116 (35%)	104 (32%)
Cardiovascular		29 (9%)	20 (6%)
**Type of surgery**			
TAH/BSO		95 (29%)	97 (29%)
TAH/BSO + LND/full staging		143 (43%)	131 (40%)
TLH/BSO		45 (14%)	41 (12%)
TLH/BSO + LND/full staging		47 (14%)	61 (18%)
**Number of nodes removed**			
TAH/BSO or TLH/BSO		0 (0–0)	0 (0–0)
TAH/BSO or TLH/BSO +LND/full staging		15 (9–25)	14 (8–22)
Missing		9	16
**Radiotherapy**			
EBRT completion		329 (100%)	325 (99%)
Dose at prescription point			
	Dose <45 Gy	1 (<1%)	4 (1%)
	Dose 45·0–50·4 Gy	329 (100%)	322 (98%)
	Dose >50·4 Gy	0	4 (1%)
Vaginal brachytherapy boost		151 (46%)	158 (48%)
**Chemotherapy completed**			
1 cycle cisplatin		326 (99%)	··
2 cycles cisplatin		304 (92%)	··
1 cycle carboplatin and paclitaxel*		302 (91%) and 302 (91%)	··
2 cycles carboplatin and paclitaxel*		294 (89%) and 291 (88%)	··
3 cycles carboplatin and paclitaxel*		279 (85%) and 263 (80%)	··
4 cycles carboplatin and paclitaxel*		262 (79%) and 233 (71%)	··

Data are median (IQR) or n (%). NCRI=National Cancer Research Institute. DGOG=Dutch Gynaecological Oncology Group. ANZGOG=Australia and New Zealand Gynaecologic Oncology Group. MaNGO=Mario Negri Gynaecologic Oncology Group. CCTG=Canadian Cancer Trials Group. FIGO=International Federation of Gynecology and Obstetrics. EEC=endometrioid endometrial cancer. LVSI=lymphovascular space invasion. TAH/BSO=total abdominal hysterectomy with bilateral salpingo-oophorectomy. LND=lymph node dissection. TLH=total laparoscopic hysterectomy. EBRT=external beam radiotherapy.

* In some cases, both drugs were not given because of toxicities.

Radiotherapy was discontinued in one patient (<1%) in the chemoradiotherapy group because of disease progression and five patients (1·5%) in the radiotherapy group because of toxicity (table 1). 329 (100%) of 330 patients in the chemoradiotherapy group and 322 (98%) of 330 patients in the radiotherapy group received an external beam pelvic radiotherapy dose between 45·0 and 50·4 Gy. Vaginal brachytherapy was given in 309 (47%) patients (151 [46%] chemoradiotherapy patients *vs* 158 [48%] radiotherapy patients). Apart from the protocol indication for brachytherapy boost (cervical invasion), 28 (4%) patients received a brachytherapy boost for locally perceived reasons such as LVSI, grade 3, or stage III.

Both cycles of concurrent cisplatin were completed by 304 (92%) of 330 patients in the chemoradiotherapy group. Adjuvant chemotherapy was started by 304 (92%) patients, while 262 (79%) patients completed all four cycles of carboplatin and 233 (71%) patients completed all four cycles of paclitaxel (table 1). At least one dose reduction of cisplatin (to 40 mg/m^2^) was recorded for five (2%) patients, of carboplatin (from AUC5 to AUC4) for 36 (11%) patients, and of paclitaxel (from 175 mg/m^2^ to 135 mg/m^2^) for 50 (15%) patients. Chemotherapy was discontinued in 61 (18%) patients; in 31 (9%) because of toxicity, patient decision in 20 (6%), disease progression in seven (2%), and for other reasons in three (1%).

Evaluation of the TROG quality assurance programme for the ANZGOG group showed that a radiotherapy benchmarking exercise before participation in the trial ensured high conformity and low rates of both minor and major contouring deviations.^16^ Evaluation of radiotherapy plans from centres in other countries is ongoing and will be reported separately.

At final database lock on May 1, 2017, 136 patients had died (61 in the chemoradiotherapy group and 75 in the radiotherapy group) and 186 patients had a failure-free survival event (83 in the chemoradiotherapy group and 103 in the radiotherapy group). Among the patients assigned to chemoradiotherapy, 50 (82%) had died from endometrial cancer, four (7%) from a second cancer, three (5%) from other intercurrent disease, and two (3%) from treatment for metastatic disease. Among the patients assigned to radiotherapy, 68 (91%) had died from endometrial cancer and five (7%) from a second cancer. For the remaining four patients (two patients treated with chemoradiotherapy and two patients with radiotherapy), the cause of death was uncertain. In one patient in the radiotherapy group, death was due to either disease progression or late treatment complications; in two patients in the chemoradiotherapy group and one in the radiotherapy group, death was due to either intercurrent disease or late treatment-related toxicity. These four deaths were counted as failure-free survival events after discussion with the DSMB.

**Figure 2:**
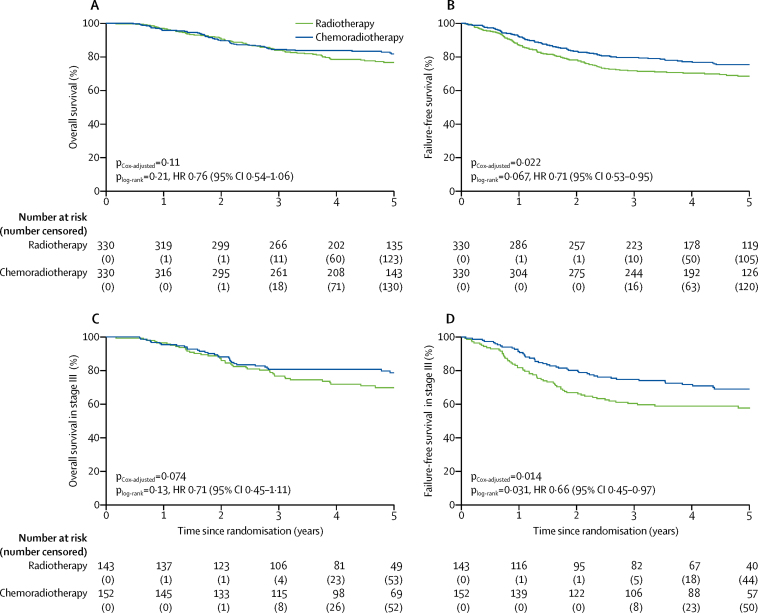
Overall survival and failure-free survival Kaplan-Meier survival curves for overall survival (A) and failure-free survival (B) in all patients, and for overall survival (C) and failure-free survival (D) of patients with stage III endometrial cancer. P_log-rank_=unadjusted log-rank p value. P_Cox adjusted_=p value adjusted for stratification factors. HR=hazard ratio.

Estimated overall survival adjusted for stratification factors at 5 years was 81·8% (95% CI 77·5–86·2) for patients in the chemoradiotherapy group versus 76·7% (72·1–81·6) for patients in the radiotherapy group (HR 0·76, 95% CI 0·54–1·06; p=0·109; table 2, figure 2). 5-year failure-free survival was 75·5% (70·3–79·9) in the chemoradiotherapy group versus 68·6% (63·1–73·4) in the radiotherapy group (HR 0·71, 0·53–0·95; p=0·022). Without adjusting for the stratification factors, the HR for overall survival was 0·81 (95% CI 0·58–1·13; p=0·213) and for failure-free survival was 0·76 (0·57–1·02; p=0·067; table 2, figure 2).

**Table 2:** Survival and recurrence outcomes

		**Events**	**5-year estimate, % (95% CI)**	**Hazard ratio (95% CI)**	**p value**
Overall survival*		··	··	0·76 (0·54–1·06)	0·109
Failure-free survival*		··	··	0·71 (0·53–0·95)	0·022
Overall survival†					
	Chemoradiotherapy	61	81·8% (77·5–86·2)	0·81 (0·58–1·13)	0·213
	Radiotherapy	75	76·7% (72·1–81·6)	··	··
Failure-free survival†					
	Chemoradiotherapy	83	75·5% (70·3–79·9)	0·76 (0·57–1·02)	0·067
	Radiotherapy	103	68·6% (63·1–73·4)	··	··
Vaginal recurrence (first recurrence)†					
	Chemoradiotherapy	1	0·3% (0·0–2·1)	0·99 (0·06–15·90)	0·999
	Radiotherapy	1	0·3% (0·0–2·1)	··	··
Pelvic recurrence (first recurrence)†					
	Chemoradiotherapy	3	1·0% (0·3–2·9)	0·60 (0·14–2·49)	0·473
	Radiotherapy	5	1·5% (0·6–3·6)	··	··
Distant metastases (first recurrence)†					
	Chemoradiotherapy	76	22·4% (18·1–27·4)	0·78 (0·58–1·06)	0·108
	Radiotherapy	93	28·3% (23·7–33·7)	··	··
Vaginal recurrence (total)†					
	Chemoradiotherapy	8	2·1% (1·0–4·4)	0·99 (0·37–2·65)	0·995
	Radiotherapy	8	2·1% (1·0–4·4)	··	··
Pelvic recurrence (total)†					
	Chemoradiotherapy	16	4·9% (3·0–7·9)	0·51 (0·28–0·92)	0·026
	Radiotherapy	31	9·2% (6·5–12·9)	··	··
Distant metastases (total)†					
	Chemoradiotherapy	79	23·1% (18·8–28·3)	0·77 (0·57–1·03)	0·077
	Radiotherapy	97	29·7% (24·9–35·1)	··	··

* Data are chemotherapy versus radiotherapy (Cox-adjusted p value), adjusted for stratification factors: participating groups, type of surgery (abdominal hysterectomy and salpingo-oophorectomy *vs* abdominal surgery plus lymphadenectomy *vs* laparoscopic procedure *vs* laparoscopic procedure plus lymphadenectomy), stage (FIGO 2009 IA *vs* IB *vs* II *vs* III), and histological type (endometrioid carcinoma *vs* serous or clear cell carcinoma).

† Log-rank p value, unadjusted for stratification factors.

In subgroup analysis, women with stage III endometrial cancer had significantly lower overall survival and failure-free survival than those with stage I–II disease (Table 3, Table 4). 5-year overall survival for stage III cancer was 78·7% (95% CI 72·2–85·7) in the chemoradiotherapy group versus 69·8% (62·4–78·1) in the radiotherapy group (HR 0·71, 95% CI 0·45–1·11; p=0·13; adjusted p=0·074). 5-year failure-free survival for stage III cancer was 69·3% (95% CI 61·1–76·2) in the chemoradiotherapy group versus 58·0% (49·3–65·7) in the radiotherapy group (HR 0·66, 95% CI 0·45–0·97; p=0·031; adjusted p=0·014; figure 2). 5-year failure-free survival for stage I–II patients was 80·8% (74·1–86·0) in the chemoradiotherapy group versus 76·6% (69·5–82·2) in the radiotherapy group (0·85, 0·54–1·33; p=0·47).

**Table 3:** Multivariable analysis of prognostic factors for overall survival

		**Patients (n)**	**Events (n)**	**5-year overall survival (95% CI)**	**Hazard ratio (95% CI)**	**p value**
Total		660	136	79% (74·8–83·9)	··	··
Treatment group		··	··	··	··	0·075
	Radiotherapy	330	75	77% (72·1–81·6)	··	··
	Chemoradiotherapy	330	61	82% (77·5–86·2)	0·73 (0·52–1·03)	··
Age (years)		··	··	··	··	<0·0001
	<60	268	31	89% (85·0–92·9)	··	··
	60–69	272	66	75% (69·6–80·6)	2·31 (1·48–3·59)	··
	≥70	120	39	67% (58·7–76·3)	3·29 (1·99–5·44)	··
Stage		··	··	··	··	<0·0001
	Stage I and II	365	59	83% (79·1–87·3)	··	··
	Stage III	295	77	74% (69·3–79·7)	2·41 (1·66–3·51)	··
Histology and grade		··	··	··	··	<0·0001
	Endometrioid grade 1 and 2	258	36	86% (81·9–90·9)	··	··
	Endometrioid grade 3	213	45	79% (73·0–85·7)	1·76 (1·10–2·81)	··
	Serous/clear cell	189	55	71% (65·2–77·4)	2·35 (1·48–3·72)	··
LVSI		··	··	··	··	0·11
	No	271	43	85% (80·5–89·4)	··	··
	Yes	389	93	75% (70·9–79·9)	1·36 (0·93–1·98)	··
Lymphadenectomy		··	··	··	··	0·33
	No	278	61	77% (71·4–82·1)	··	··
	Yes	382	75	81% (77·1–85·2)	0·82 (0·55–1·22)	··

Adjusted for participating groups. LVSI=lymph-vascular space invasion.

**Table 4:** Multivariable analysis of prognostic factors for failure-free survival

		**Patients (n)**	**Events (n)**	**5-year failure-free survival (95% CI)**	**Hazard ratio (95% CI)**	**p value**
Total		660	186	72% (66·7–76·7)	··	··
Treatment group		··	··	··	··	0·010
	Radiotherapy	330	103	68% (63·1–73·4)	··	··
	Chemoradiotherapy	330	83	75% (70·3–79·9)	0·68 (0·51–0·91)	··
Age (years)		··	··	··	··	<0·0001
	<60	268	54	81% (75·3–85·0)	··	··
	60–69	272	87	67% (60·7–72·4)	1·74 (1·23–2·46)	··
	≥70	120	45	64% (54·4–71·7)	2·14 (1·41–3·25)	··
Stage		··	··	··	··	<0·0001
	Stage I and II	365	78	79% (73·9–82·6)	··	··
	Stage III	295	108	64% (58·0–69·2)	2·62 (1·90–3·61)	··
Histology and grade		··	··	··	··	<0·0001
	Endometrioid grade 1 and 2	258	58	78% (72·7–83·1)	··	··
	Endometrioid grade 3	213	60	71% (64·5–77·1)	1·56 (1·06–2·30)	··
	Serous or clear cell	189	68	64% (56·6–70·4)	2·15 (1·46–3·16)	··
LVSI		··	··	··	··	0·054
	No	271	62	77% (71·4–81·8)	··	··
	Yes	389	124	68% (63·4–72·9)	1·36 (0·99–1·87)	··
Lymphadenectomy		··	··	··	··	0·41
	No	278	81	72% (65·7–76·6)	··	··
	Yes	382	105	72% (67·4–76·7)	0·87 (0·61–1·22)	··

Adjusted for participating groups. LVSI=lymph-vascular space invasion.

Serous cancers (>25% serous component) had significantly lower overall survival and failure-free survival than the other histological subtypes; failure-free was 58% (95% CI 42–70) with chemoradiotherapy versus 48% (34–61) with radiotherapy (HR 0·63, 95% CI 0·36–1·12; p=0·11). The number of patients and events are, however, small in these subgroups (appendix p 3).

Isolated vaginal and pelvic recurrences were rare, with isolated vaginal recurrence diagnosed in one (<1%) patient in the chemoradiotherapy group and in one (<1%) patient in the radiotherapy group (p=0·995), and isolated pelvic recurrence in three (1%) patients in the chemoradiotherapy group versus five (2%) patients in the radiotherapy group (p=0·473). Most recurrences were distant metastases: 76 (22%) patients in the chemoradiotherapy group versus 93 (28%) patients in the radiotherapy group were diagnosed with distant metastases (p=0·108). The 5-year estimate of pelvic recurrence (both isolated and combined pelvic and distant recurrences) was 4·9% (95% CI 3·0–7·9) for the chemoradiotherapy group versus 9·2% (6·5–12·9) for the radiotherapy group (p=0·026; table 2).

In the multivariable analysis, the following covariates were included together with treatment: stage, histological type and grade, type of surgery, participating groups, LVSI, and age. In the presence of these factors, combined chemotherapy and radiotherapy significantly improved failure-free survival. Most factors, except lymphadenectomy, were significantly correlated with failure-free survival (table 4).

**Figure 3:**
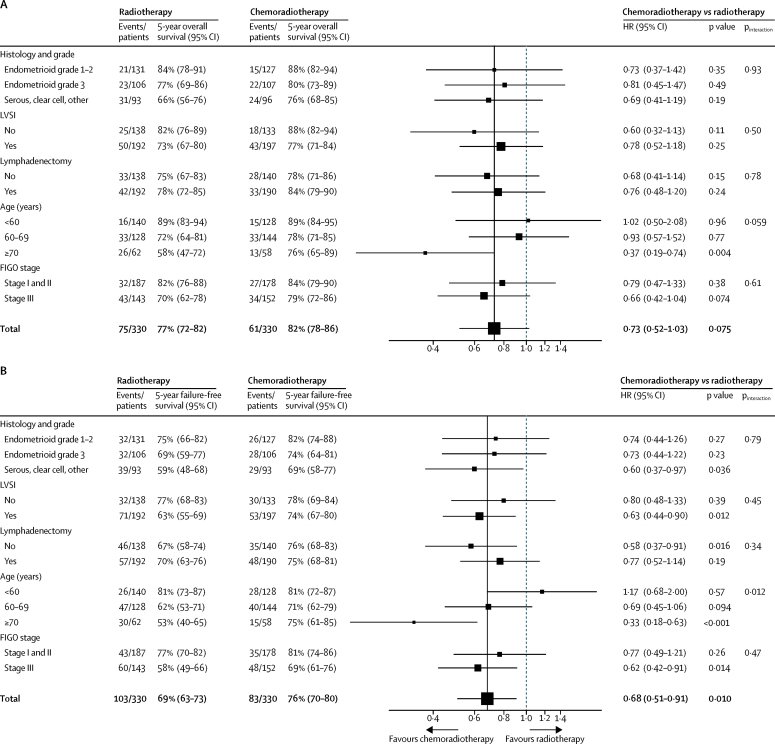
Forest plot of multivariable analysis (treatment by covariate interaction) of overall survival (A) and failure-free survival (B) For the multivariable analysis the stratification factors (participating group, lymphadenectomy, stage of cancer, and histological type), lymphovascular space invasion, and age were used. HR=hazard ratio. LVSI=lymphovascular space invasion. FIGO=International Federation of Gynecology and Obstetrics.

In multivariable analysis for failure-free survival, only age group was found to be predictive of treatment effect, with a strong treatment-by-age effect (p_interaction_=0·012, figure 3). Women aged 70 years or older had the greatest benefit from chemoradiotherapy.

Grade 2 or worse adverse events were reported during treatment in 308 (93%) women in the chemoradiotherapy group versus 144 (43%) in the radiotherapy group, and grade 3 or worse in 198 (60%) versus 41 (12%; p<0·0001; table 5); the majority of grade 3 or worse adverse events were haematological. Table 6 shows an overview of adverse events at 6 months after randomisation, which was about 1 month after completion of treatment in the chemoradiotherapy group. There were no treatment-related deaths. From 12 months onwards, no significant differences between the groups were found in grade 3 or worse adverse events (appendix p 5). The number of patients with grade 2 or worse adverse events was 86 (32%) for chemoradiotherapy versus 64 (24%) for radiotherapy at 3 years (p=0·034), and 57 (40%) versus 38 (28%) at 5 years (p=0·033). The most significant and clinically relevant difference between the arms was found for grade 2 or worse sensory neuropathy, which persisted in 20 (8%) women in the chemoradiotherapy group versus one (1%) women in the radiotherapy group at 3 years and 12 (9%) women versus no women at 5 years (both p<0·0001). An extensive overview of adverse events during follow-up is in the appendix (pp 4–5).

**Table 5:** Adverse events reported during treatment

		**Grade 2**			**Grade 3–4**		
		Chemoradiotherapy	Radiotherapy	p value*	Chemoradiotherapy	Radiotherapy	p value†
Any		110 (33%)	103 (31%)	<0·0001	198 (60%)	41 (12%)	<0·0001
Any grade 3		NA	NA	··	148 (45%)	41 (12%)	··
Any grade 4		NA	NA	··	50 (15%)	0	··
Auditory or hearing		14 (4%)	3 (1%)	0·011	1 (<1%)	1 (<1%)	1·00
Allergy		23 (7%)	1 (<1%)	<0·0001	5 (2%)	0	0·062
Fatigue		69 (21%)	7 (2%)	<0·0001	10 (3%)	0	0·0018
Hypertension		19 (6%)	12 (4%)	0·14	6 (2%)	3 (1%)	0·50
Alopecia		187 (57%)	1 (<1%)	<0·0001	NA	NA	··
Dermatitis		18 (5%)	5 (2%)	0·013	1 (<1%)	1 (<1%)	1·0
Any gastrointestinal		145 (44%)	79 (24%)	<0·0001	47 (14%)	18 (5%)	<0·0001
	Diarrhoea	104 (32%)	69 (21%)	<0·0001	35 (11%)	14 (4%)	0·0027
	Nausea	68 (21%)	24 (7%)	0·0010	9 (3%)	2 (1%)	0·06
	Vomiting	31 (9%)	9 (3%)	<0·0001	5 (2%)	0	0·06
	Anorexia	30 (9%)	9 (3%)	0·0033	3 (1%)	4 (1%)	1·00
	Constipation	32 (10%)	6 (2%)	<0·0001	1 (<1%)	0	1·00
Genito-urinary: frequency or urgency		24 (7%)	10 (3%)	0·020	2 (1%)	2 (1%)	1·00
Any haematological		100 (30%)	19 (6%)	<0·0001	149 (45%)	18 (5%)	<0·0001
	Febrile neutropenia	NA	NA	··	9 (3%)	1 (<1%)	0·021
	Infection with neutropenia	3 (1%)	0	0·0018	7 (2%)	0	0·015
	Infection without neutropenia	21 (6%)	1 (<1%)	<0·0001	12 (4%)	1 (<1%)	<0·0001
	Haemoglobin	105 (32%)	0	<0·0001	27 (8%)	0	<0·0001
	Leucocytes	98 (30%)	3 (1%)	<0·0001	76 (23%)	1 (<1%)	<0·0001
	Lymphocytes	48 (15%)	16 (5%)	<0·0001	109 (33%)	17 (5%)	<0·0001
	Neutrophils	62 (19%)	1 (<1%)	<0·0001	66 (20%)	1 (<1%)	<0·0001
	Platelets	22 (7%)	0	<0·0001	18 (5%)	0	<0·0001
Metabolic or laboratory		15 (5%)	1 (<1%)	<0·0001	3 (1%)	0	0·25
Any neuropathy		82 (25%)	1 (<1%)	<0·0001	23 (7%)	0	<0·0001
	Motor	13 (4%)	1 (<1%)	<0·0001	4 (1%)	0	0·12
	Sensory	79 (24%)	0	<0·0001	22 (7%)	0	<0·0001
Any pain		101 (31%)	23 (7%)	<0·0001	31 (9%)	4 (1%)	<0·0001
	Joint	52 (16%)	2 (1%)	<0·0001	10 (3%)	0	0·0018
	Muscle	52 (16%)	1 (<1%)	<0·0001	9 (3%)	0	0·0037
	Pelvic, back, or limb	10 (3%)	4 (1%)	<0·0001	11 (3%)	0	<0·0001
Pulmonary: dyspnoea		12 (4%)	2 (1%)	<0·0001	5 (2%)	0	0·062
Thrombosis or embolism		2 (1%)	0	0·031	4 (1%)	0	0·12

Data are n (%). Adverse events are listed that occurred in at least 5% of patients, or were significantly different between the study groups at any timepoint during treatment, or both. Adverse events were calculated at each timepoint. For each adverse event, the maximum grade per patient was calculated (worst ever by patient). Adverse events were graded according to Common Terminology Criteria for Adverse Events version 3.0. Chemoradiotherapy, n=330; radiotherapy, n=330. NA=not applicable.

* Significance level for grade 2, 3, and 4.

† Significance level for grade 3 and 4.

**Table 6:** Adverse events reported at 6 months after randomisation

		**Grade 2**			**Grade 3–4**		
		Chemoradiotherapy	Radiotherapy	p value*	Chemoradiotherapy	Radiotherapy	p value†
Any		128 (39%)	96 (29%)	<0·0001	54 (16%)	25 (8%)	<0·0001
Any grade 3		NA	NA	··	49 (15%)	21 (6%)	··
Any grade 4		NA	NA	··	5 (2%)	4 (1%)	··
Auditory or hearing		8 (2%)	3 (1%)	0·22	0	0	1·00
Allergy		2 (1%)	1 (<1%)	1·00	0	0	1·00
Fatigue		10 (3%)	2 (1%)	0·054	1 (<1%)	1 (<1%)	1·00
Hypertension		15 (5%)	18 (5%)	0·75	5 (2%)	5 (2%)	1·00
Alopecia		64 (19%)	0	<0·0001	NA	NA	··
Dermatitis		1 (<1%)	0	1·00	0	0	1·00
Any gastrointestinal		19 (6%)	18 (5%)	0·89	7 (2%)	9 (3%)	0·80
	Diarrhoea	8 (2%)	11 (3%)	0·20	0	3 (1%)	0·25
	Nausea	7 (2%)	5 (2%)	0·35	5 (2%)	2 (1%)	0·45
	Vomiting	7 (2%)	6 (2%)	0·45	3 (1%)	0	0·25
	Anorexia	1 (<1%)	4 (1%)	0·50	2 (1%)	2 (1%)	1·00
	Constipation	7 (2%)	4 (1%)	0·79	1 (<1%)	2 (1%)	1·00
Genito-urinary: frequency or urgency		5 (2%)	6 (2%)	0·77	1 (<1%)	0	1·00
Any haematological		54 (16%)	27 (8%)	<0·0001	24 (7%)	6 (2%)	0·0001
	Febrile neutropenia	NA	NA	··	0	0	1·00
	Infection with neutropenia	0	0	1·00	1 (<1%)	0	1·00
	Infection without neutropenia	5 (2%)	1 (<1%)	0·22	0	0	1·00
	Haemoglobin	24 (7%)	0	<0·0001	3 (1%)	0	0·25
	Leucocytes	20 (6%)	1 (<1%)	<0·0001	6 (2%)	1 (<1%)	0·12
	Lymphocytes	43 (13%)	26 (8%)	0·0015	17 (5%)	5 (2%)	0·015
	Neutrophils	4 (1%)	0	0·0018	6 (2%)	0	0·031
	Platelets	5 (2%)	0	0·015	2 (1%)	0	0·50
Metabolic or laboratory		2 (1%)	0	0·12	2 (1%)	0	0·50
Any neuropathy		42 (13%)	1 (<1%)	<0·0001	8 (2%)	2 (1%)	0·11
	Motor	7 (2%)	1 (<1%)	0·09	3 (1%)	2 (1%)	1·00
	Sensory	41 (12%)	0	<0·0001	6 (2%)	0	0·031
Any pain		31 (9%)	32 (10%)	0·54	3 (1%)	7 (2%)	0·34
	Joint	8 (2%)	6 (2%)	1·00	0	1 (<1%)	1·00
	Muscle	5 (2%)	1 (<1%)	0·22	0	0	1·00
	Pelvic, back, or limb	10 (3%)	9 (3%)	0·33	0	3 (1%)	0·25
Pulmonary: dyspnoea		1 (<1%)	0	1·00	1 (<1%)	1 (<1%)	1·00
Thrombosis or embolism		3 (1%)	0	0·37	1 (<1%)	1 (<1%)	1·00

Data are n (%). Adverse events are listed that occurred in at least 5% of patients, or were significantly different between the study groups at any timepoint, or both. Adverse events were calculated at each timepoint. For each adverse event, the maximum grade per patient was calculated (worst ever by patient). Adverse events were graded according to Common Terminology Criteria for Adverse Events version 3.0. Chemoradiotherapy, n=329; radiotherapy, n=329. NA=not applicable.

* Significance level for grade 2, 3, and 4.

† Significance level for grade 3 and 4.

## Discussion

The final results of the PORTEC-3 trial showed that the combination of adjuvant chemotherapy and radiotherapy for high-risk endometrial cancer did not significantly improve overall survival. However, chemoradiotherapy did improve 5-year failure-free survival compared with radiotherapy alone. Patients with stage III disease—who had a higher risk of recurrence than those with stages I–II—had a HR of 0·66 and 11% absolute improvement of failure-free survival with chemo-radiotherapy, which is clinically relevant and exceeds the 10% improvement used when designing the study.

The improvement in failure-free survival in the chemoradiotherapy group should be weighed against the severity and duration of toxicity of combined treatment, especially since overall survival was not significantly improved. Although significantly higher incidences of adverse events and reduced health-related quality of life were reported in the chemoradiotherapy group during and after treatment,^9^ rapid recovery was seen, with no differences in grade 3–4 adverse events from 12 months onwards. Grade 2 sensory neuropathy, however, persisted significantly more often in patients treated with chemoradiotherapy, with 25% of patients reporting “quite a bit” or “very much” tingling or numbness at 2 years, compared with 6% for radiotherapy.^9^ Sensory neuropathy is associated with lower levels of functioning and quality of life, and more fatigue.^24^

For decades, standard adjuvant treatment for women with high-risk endometrial cancer has been pelvic external beam radiotherapy. It has been hypothesised that chemotherapy might improve survival by reducing the risk of metastatic disease. Randomised trials comparing adjuvant chemotherapy with external beam radiotherapy failed to show an improvement in progression-free survival or overall survival.^6^, ^7^ Retrospective studies reported substantial rates of pelvic recurrence if high-risk patients were treated without radiotherapy, supporting the combined use of pelvic radiotherapy with adjuvant chemotherapy, as first explored in the RTOG 9708 phase 2 trial.^8^, ^25^, ^26^

Randomised studies have compared radiotherapy with the combination of chemotherapy and radiotherapy in patients with high-risk endometrial cancer. The NSGO-EC-9501/EORTC-55991 trial compared external beam radiotherapy alone with external beam radiotherapy and four cycles of platinum-based chemotherapy, given sequentially before or after external beam radiotherapy. A pooled analysis with the ManGO Iliade 3 trial^27^ with a total cohort of 534 patients showed, in line with our results, improved progression-free survival (78% *vs* 69%, p=0·01) and a trend for improved survival (82% *vs* 75%, p=0·07) with the addition of chemotherapy to radiotherapy alone.

The schedule of combined radiotherapy with concurrent and adjuvant chemotherapy used in the PORTEC-3 trial seemed likely to be most effective because both treatments were started early after surgery and thus maximum benefit of the combination could be expected. In RTOG-9708, 4-year overall survival was 85% and disease-free survival was 81%. A retrospective single institution study^28^ reporting on 40 patients with stage IIIA or IIIC endometrial cancer treated with the same combination of chemoradiotherapy showed 5-year overall survival of 85% and relapse-free survival of 79%. In the chemoradiotherapy group of the PORTEC-3 trial the 5-year overall survival probability was 82% and the failure-free survival probability was 76%, thus confirming these results in a much larger trial. Overall and relapse-free survival in the pooled NSGO-EC-9501/EORTC-55991/Iliade trials^27^ were also similar at 82% and 78%, with only 20% patients with stage III disease.

High-risk endometrial cancer is heterogeneous, including various histological types and stages of disease. In the NSGO-EC-9501/EORTC-55991 trial, although progression-free survival was significantly improved for patients with endometrioid endometrial cancer, this improvement was not found for serous and clear cell cancers. A Gynecologic Oncology Group (GOG) study^29^ explored the associations between histology and outcome in advanced or recurrent endometrial cancer patients in chemotherapy trials in 1203 patients. Although serous and clear cell cancers had a worse prognosis than other histological types, no difference in benefit from chemotherapy was found. In the PORTEC-3 trial, women with serous or clear-cell cancers had at least as much improvement in failure-free survival with the addition of chemotherapy as women with endometriod endometrial cancer did. When comparing serous cancers with other histological types, as expected, worse overall survival and failure-free survival were found for serous cancers; patients with serous cancers had a failure-free survival benefit with chemoradiotherapy, but this benefit was not significant in view of the small numbers of serous cancers and events.

The multivariable analysis indicated that women older than 70 years seemed to have a greater failure-free survival benefit from chemotherapy than younger women. Age is a well-known risk factor for endometrial cancer and a greater benefit of chemotherapy in older women has been reported previously.^7^, ^30^ Although selection of fitter older women in this randomised trial might have occurred, physicians should not be reticent to counsel older women about the possible benefits of combined chemotherapy and radiotherapy.

Analysed by stage, patients with stage III endometrial cancer who have the highest frequency of recurrence, also had the greatest absolute benefit from the combined treatment. The smaller failure-free survival improvement for patients with stage I–II disease seems not to outweigh the cost in terms of toxicity and quality-of-life impairment. Pelvic control was high (91%) with radiotherapy alone. This finding is in line with the results of the GOG-249 trial, in which patients with stage I and II endometrial cancer with high-intermediate or high-risk factors were randomly assigned to pelvic radiotherapy alone or to chemotherapy (three cycles of carboplatin and paclitaxel) followed by vaginal brachytherapy. No superiority of three cycles chemotherapy plus vaginal brachytherapy over external bean radiotherapy alone was found, with overlapping progression-free and overall survival curves and significantly more pelvic and para-aortic recurrences in the chemotherapy group.^31^, ^32^

In 47% of all patients, a vaginal brachytherapy boost was given (46% for chemoradiotherapy *vs* 48% for radiotherapy); the majority because of cervical involvement and 4% because of other reasons, such as LVSI or grade 3 endometrial cancer. This finding is in line with other studies among patients with stage II–III endometrial cancer.^33^ Although the addition of a brachytherapy boost might have added to the good local control seen in both groups, we do not expect this would have affected the results, because the proportion of women receiving a brachytherapy boost was equal in the two treatment groups.

To compare the radiotherapy and chemotherapy schedule as used in the PORTEC-3 trial with chemotherapy alone for advanced stage endometrial cancer, the GOG-258 trial randomly assigned participants to receive chemoradiotherapy or six cycles of carboplatin and paclitaxel.^34^ Final results are pending, but a presented abstract^34^ reported no differences in overall or recurrence-free survival, while significantly more vaginal and pelvic or para-aortic recurrences were reported in patients treated with chemotherapy alone. Similar results were also reported in a retrospective multicentre study^35^ of 265 patients with stage IIIC disease treated with chemotherapy, radiotherapy, or both. Patients treated with chemotherapy alone were two to seven times more likely to develop a vaginal recurrence (35%) than those treated with radiotherapy (18%) or chemoradiotherapy (5%), and twice as likely to develop an isolated pelvic recurrence (18% *vs* 9% *vs* 7%). These outcomes confirm the importance of combined radiotherapy and chemotherapy to maximise vaginal and pelvic control and relapse-free survival. Furthermore, acute gastrointestinal and genitourinary toxicity of pelvic radiotherapy will be reduced with the current standard use of intensity-modulated radiotherapy.^36^

PORTEC-3 was a multicentre trial with strong international collaboration among six participating groups and, therefore, highly representative of current practice worldwide. Upfront pathology review was done to include only truly high-risk patients in the trial. Analysis of pathology review in the Netherlands and the UK (48% of PORTEC-3 participants) revealed that 8·3% of patients did not fulfil the eligibility criteria after central pathology review. These patients did not enter the trial.^15^

A limitation of this trial might be that because of the death and failure-free survival event rates were lower than expected at the time of trial design, the required number of overall survival events was not reached and the final analysis was time-based rather than event-based, with final analysis at a median follow-up of 5 years (42 months after inclusion of the last patient). The number of overall survival events was 136 (69% of the required number of overall survival events), and the number of failure-free survival events was 186 (94% of the required events). The non-significant difference in 5-year overall survival of 5% found in PORTEC-3 was smaller than the study was powered to detect, and overall survival and failure-free survival probabilities were higher than expected from previous studies. Long-term outcomes will be analysed, especially for overall survival.

The costs of chemoradiotherapy in terms of toxicity and treatment duration should be weighed against the benefits, and this cost–benefit tradeoff could be seen differently from the patient or physician perspective. In a patient preference study done by the ANZGOG group among PORTEC-3 participants,^37^ more than 50% of patients rated 5% survival improvement sufficient to make chemotherapy worthwhile. Although the trial results are in the range of this benefit for failure-free survival, the overall survival difference was not significant, thus individual patient counselling remains essential. Translational studies of molecular risk factors and tumour characteristics with the tumour samples of the PORTEC-3 participants might identify those who could most benefit from chemotherapy or targeted agents and individualise treatment of women with high-risk endometrial cancer.^38^

In conclusion, although treatment with chemoradiotherapy significantly improved 5-year failure-free survival for patients with high-risk endometrial cancer compared with radiotherapy alone, there was no significant difference in overall survival. For women with stage III endometrial cancer, a significant improvement in failure-free survival was found. For each patient, the cost in terms of increased toxicity and longer treatment duration should be weighed against the benefit in terms of improvement in failure-free survival. Because pelvic control was high with radiotherapy alone, this chemoradiotherapy schedule cannot be recommended as a new standard for patients with stage I–II endometrial cancer. However, in view of the higher risk of recurrence among women with stage III disease, this chemoradiotherapy schedule should be considered to maximise failure-free survival, and benefits and risks should be individually discussed.

For the **study protocol** see http://www.msbi.nl/portec3

**This online publication has been corrected. The corrected version first appeared at thelancet.com/oncology on March 28, 2018**
