# Supplementary material for: Adjuvant chemoradiotherapy versus radiotherapy alone for women with high-risk endometrial cancer (PORTEC-3): final results of an international, open-label, multicentre, randomised, phase 3 trial
Source: Lancet Oncol. 2018 Mar;19(3):295–309. doi: 10.1016/S1470-2045(18)30079-2 (PMC5840256; doi:10.1016/S1470-2045(18)30079-2)
Supplement: Supplementary appendix [file mmc1.pdf]

THE LANCET
Oncology

Supplementary appendix

This appendix formed part of the original submission and has been peer reviewed. We post it as supplied by the authors.

Supplement to: de Boer SM, Powell ME, Mileshkin L, et al, on behalf of the PORTEC study group. Adjuvant chemoradiotherapy versus radiotherapy alone for women with high-risk endometrial cancer (PORTEC-3): final results of an international, open-label, multicentre, randomised, phase 3 trial. *Lancet Oncol* 2018; published online Feb 12. http://dx.doi.org/10.1016/S1470-2045(18)30079-2.

PORTEC 3 Study group and participating centres

PORTEC-3 Independent data monitoring committee:
L.V.A.M. Beex, N. James, M.J.M. Olofsen-van Acht, W. Parulekar, W.L.J. van Putten, D. Rischin, J. Yarnold (chair)
PORTEC-3 trial statistician: H. Putter

List of participating countries and centres (listed in order of patients recruited):
United Kingdom: 184 patients (177 evaluable)
Study Coordinators: M. Powell (P.I.), London; H. Kitchener, Manchester; J. Ledermann, London
Group coordinating trial centre: Cancer Research UK and UCL Cancer Trials Centre, London
Trial pathologists: N. Singh, London; G. Wilson, Manchester
Participating centres and principal investigator(s) (number of patients):
London-Barts Health NHS Trust (M. Powell, 23); London-University College London Hospitals NHS Foundation Trust (M. McCormack, 15); Bebington, Wirral-The Clatterbridge Cancer Centre NHS Foundation Trust (K. Whitmarsh, K. Hyatt, 12); Wolverhampton-The Royal Wolverhampton NHS Trust (R. Allerton, 11); Cambridge-Cambridge University Hospitals NHS Foundation Trust (L.T. Tan, M. Iddawela, D. Gregory, S. Ayres, 11); Leicester-Leicester Royal Infirmary (P. Symonds, 10); Northwood-Mount Vernon Cancer Centre (P. Hoskin, 10); Middlesborough-The James Cook University Hospital (A. Rathmell, M. Adusumalli, 9); Nottingham-Nottingham City Hospital (S. Chan, A Anand, 9); Norwich-Norfolk and Norwich University Hospitals NHS Foundation Trust (R. Wade, 8); Guildford-Royal Surrey County Hospital (A. Stewart, 6); Newcastle Upon Tyne-Freeman Hospital (W. Taylor, 6); Brighton-Brighton & Sussex University Hospitals NHS Trust (K. Lankester, 5); Coventry-University Hospital Coventry (C. Irwin, M. Hocking, 5); Taunton-Musgrove Park Hospital (P. Jankowska, D. Milliken, C. Barlow, 5); Cheltenham-Cheltenham General Hospital (A. Cook, R. Counsell, 4); Exeter-Royal Devon & Exeter Hospital (P. Bliss, A. Hong, 4); Lincoln-Lincoln County Hospital (M. Panades, 4); Romford-Queens Hospital (M. Quigley, 4); Manchester-The Christie NHS Foundtion Trust (S. Davidson, 3); Northampton-Northampton General Hospital NHS Trust (C. Mak, 3); Preston-Royal Preston Hospital (A. Hindley, 3); Truro-Royal Cornwall Hospitals NHS Trust (A. Thomson, 3); Sheffield-Weston Park Hospital (S. Pledge, J. Martin, 2); Shrewsbury-Royal Shrewsbury Hospital (S. Awwad, A. Zachariah, 2); Carlisle-North Cumbria University Hospitals NHS Trust (S. Singhal, 1); Colchester-Essex County Hospital (A. Lamont, 1); London-Guy's and St. Thomas' NHS Foundation Trust (A. Winship, A. Montes, V. Mullassery, 1); London-Hammersmith Hospital - Imperial College Healthcare NHS Trust (A. Taylor, 1); Poole-Poole Hospital NHS Foundation Trust (V. Laurence, M. Flubacher, 1); Reading-Royal Berkshire Hospital (H. O'Donnell, 1); Stoke-on-Trent-Royal Stoke University Hospital (R. Bhana, S. Lupton, 1)

The Netherlands: 145 patients (138 evaluable)
Study Coordinators: C.L. Creutzberg (C.I.), Leiden; R. Kruitwagen, Maastricht; H. Nijman, Groningen; N. Ottevanger, Nijmegen
Group coordinating trial centre: Netherlands Comprehensive Cancer Organisation (IKNL), Leiden
Trial pathologists: H. Hollema, Groningen; V.T. Smit, Leiden
Participating centres and principal investigator(s) (number of patients):
University Medical Center Utrecht (I.M. Jurgenliemk-Schulz, 20); Maastro Clinic, Maastricht (L.C.H.W. Lutgens, 17); University Medical Center Groningen (E. Pras, 15); Leiden University Medical Center (C.L. Creutzberg, R. Nout, 15); University Medical Center Radboud, Nijmegen (J.W.H. Leer, A. Snyers, 11); Academic Medical Center, Amsterdam (A.L.J. Uitterhoeve, L. Stalpers, 9); Medical Spectre Twente, Enschede (J.J. Jobsen, 9); Radiotherapy institute Friesland, Leeuwarden (A. Slot, 9); Erasmus Medical Center Rotterdam (J.W.M. Mens, 8); Medical Center Haaglanden/ Radiotherapy Centre West (T.C. Stam, P.C.M. Koper, 7); Netherlands Cancer Institute, Amsterdam (B. van Triest, 6); Radiotherapy Group, Arnhem (E.M. van der Steen-Banasik, 6); Radiotherapy Institute Verbeeten, Tilburg (K.A.J. de Winter, 6); Radiotherapy Group, Deventer (S. van de Pol, 3); Catharina Hospital, Eindhoven (H.A. van den Berg, 3); VU Medical Centre, Amsterdam (O.W.M. Meijer, 1)

Australia & New Zealand: 122 patients (118 evaluable)
ANZGOG Study Coordinators: L. Mileshkin (P.I.) Melbourne; M. Quinn, Melbourne; P. Khaw Melbourne; I.Kolodziej, Sydney

Group coordinating trial centre: NHMRC Clinical Trials Centre, Sydney
Trial pathologist: J.Pyman, Melbourne
Participating centres and principal investigator(s) (number of patients):
Peter MacCallum Cancer Centre, Melbourne, Victoria, Australia (L. Mileshkin, P.Khaw; 31); Monash Cancer Centre (Monash Medical Centre), East Bentleigh, Victoria, Australia (P. Khaw/G. Goss, 20); Westmead Hospital, Wentworthville, NSW, Australia (G. Wain, 15); Auckland City Hospital , Auckland, New Zealand (S. Brooks, 13); Wellington Blood & Cancer Centre, Wellington, New Zealand (C. Johnson, 11); Calvary Mater Newcastle, Newcastle, Australia (A. Capp, 8); Christchurch Hospital, Canterbury, New Zealand (M. Vaughan, 4); Royal Hobart Hospital, Hobart, Tasmania, Australia (P. Blomfield, 3); Palmerston North Hospital, Palmerston North, New Zealand (C. Hardie, 3); Royal North Shore Hospital, St Leonards, NSW, Australia (M. Stevens, 3); Waikato Hospital, Hamilton, New Zealand (M. Kuper, 2); Royal Brisbane &Women's Hospital, Brisbane, QLD, Australia(R. Cheuk, 2); Liverpool Hospital, Liverpool, NSW, Australia (S. Vinod, 2); Mater Hospital Brisbane , South Brisbane, QLD, Australia(C. Shannon/J. Ramsay, 2); Wollongong Hospital, Wollongong, NSW, Australia (A. Glasgow, 2); Townsville Hospital, Townsville, QLD, Australia (S. Hewitt, 1)

Italy: 103 patients (98 evaluable)
MaNGO Study Coordinators: R. Fossati (P.I.) Milano; D. Katsaros, Torino; A. Colombo, Lecco
Group coordinating trial centre: Istituto di Ricerche Farmacologiche Mario Negri, Milano
Trial pathologists: S. Carinelli, Milano; C. Di Tonno, Milano
Participating centres and principal investigator(s) (number of patients):
Torino - S. Anna Hospital (S. Gribaudo, M. Mitidieri, 33); Lecco - Ospedale A. Manzoni (R. D'Amico, 24); Monza - S. Gerardo Hospital (S. Meregalli, A. A. Lissoni, 16); Torino - Ospedale Umberto I (A. Ferrero, 7); Padova – Istituto Oncologico Veneto / Mirano, Venezia – Azienda ULSS 13 (L. Corti, G. Artioli, 6); Varese - H. Del Ponte University of Insubria (C. Apolloni, 4); Ravenna - Ospedale S. Maria delle Croci (D. Turci, 4); Brescia – Spedali Civili (G. Tognon, 2); Como – ASST Lariana Ospedale S. Anna (E. Bianchi, 2); Meldola - Istituto Scientifico Romagnolo per lo Studio e la Cura dei Tumori (E. Bianchi, 2); Genova – IRCCS San Martino IST (M. Bruzzone, 1); Milano - ASST Grande Ospedale Metropolitano Niguarda (S. Siena, 1); Palermo – AOR Villa Sofia-Cervello (N. Varsellona, 1)

Canada: 65 patients (65 evaluable)
CCTG Study Coordinators: A. Fyles Toronto, Ontario; P. Bessette, Sherbrooke, Quebec
Group coordinating trial centre: Canadian Cancer Trials Group, Kingston, Ontario
Trial pathologist: M. McLachlin, London, Ontario
Participating centres and principal investigator(s) (number of patients):
Sherbrooke-Centre Hosp. Universitaire de Sherbrooke (P. Bessette, 18); Montreal-Hopital Notre-Dame de Montreal (D. Provencher, 11); Calgary-Tom Baker Cancer Centre (P. Ghatage, 9); Halifax-Queen Elizabeth II Health Sciences Centr (P. Rittenberg, 8); Montreal-McGill Oncology Montreal (L. Souhami, 7); Toronto-Sunnybrook Health Sciences Centre (G. Thomas, 7); Quebec-Hotel-Dieu de Quebec (M. Plante, 2); London-London Health Sciences Centre (A. Hammond, 1); St John's-Dr. H. Bliss Murphy Cancer Centre (P. Power, 1); Toronto-Princess Margaret Hospital (A. Fyles, 1)

France: 67 patients (64 evaluable)
FEDEGYN Study Coordinator: Chr. Haie-Meder (P.I.) Paris
Group coordinating trial centre: UNICANCER, Paris
Trial pathologist: P. Duvillard, Paris
Participating centres and principal investigator(s) (number of patients):
Besancon-Hopital Jean Minjoz (M-H Baron, 10); Rouen-Centre Henri Becquerel (Hanzen, 9); Saint Herblain-Centre Rene Gauducheau (D. Berton-Rigaud, 8); Limoges-CHU Limoges (Pr. N. Tubiana-Mathieu, 6); Bordeaux-Institut Bergonie (L. Thomas, 5); Reims-Institut Jean Godinot (A. Savoye, S. Maillard, 5); Dijon-Centre Georges Francois Leclerc (K. Peignaux, 4); Paris/ Villejuif-Institut Gustave Roussy (C. Haie Meder, 4); Clermont-Ferrand-Centre Jean Perrin (C. Benoit, 3); Montpellier-Centre Val d'Aurelle (C. Kerr, 3); Toulouse Cedex-Institut Claudius Regaud (L. Gladieff, 3); Caen-Centre Francois Baclesse (D. Lerouge, 2); Nice Cedex-Centre Antoine Lacassagne (P. Follana, 2); Marseille-Institut Paoli Calmettes (M. Cappiello, 1); Strasbourg-Centre Paul Strauss (T. Petit, 1); Tours Cedex-CHU de Tours - Hopital Bretonneau (I. Barillot, 1)

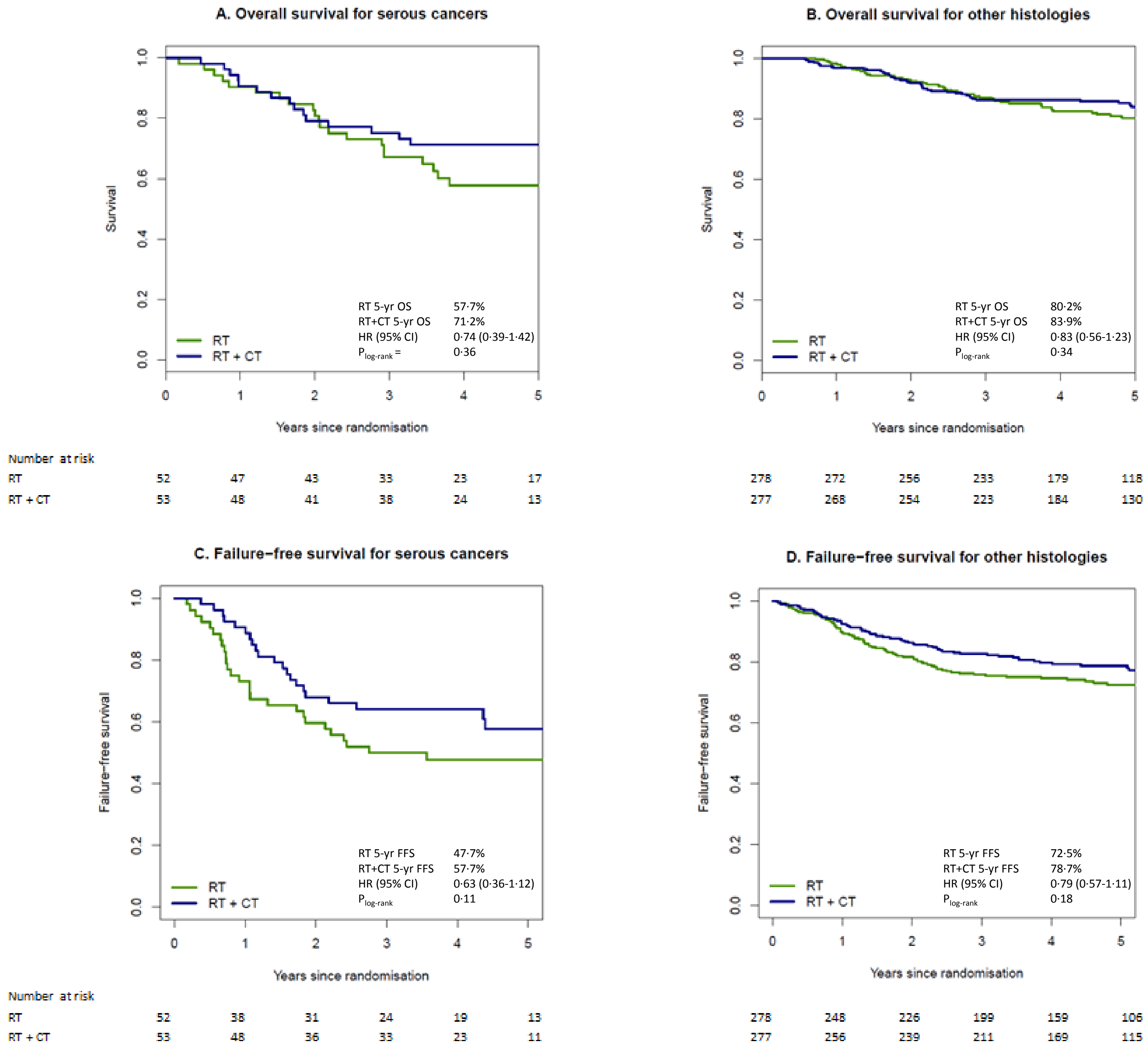

Figure S1: Kaplan-Meier survival curve for overall survival for serous cancers (A) and other histologies (B) and failure free survival curve for serous cancers (C) and other histologies (D).

A. Chemoradiotherapy

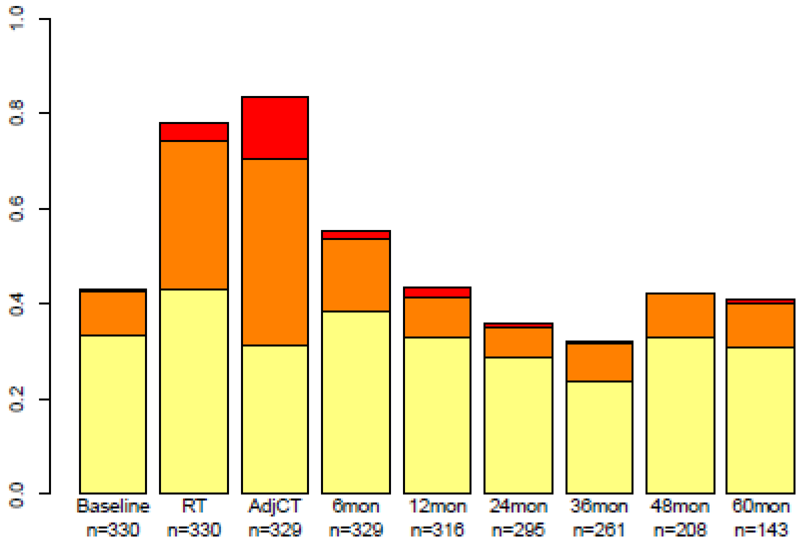

B. Radiotherapy

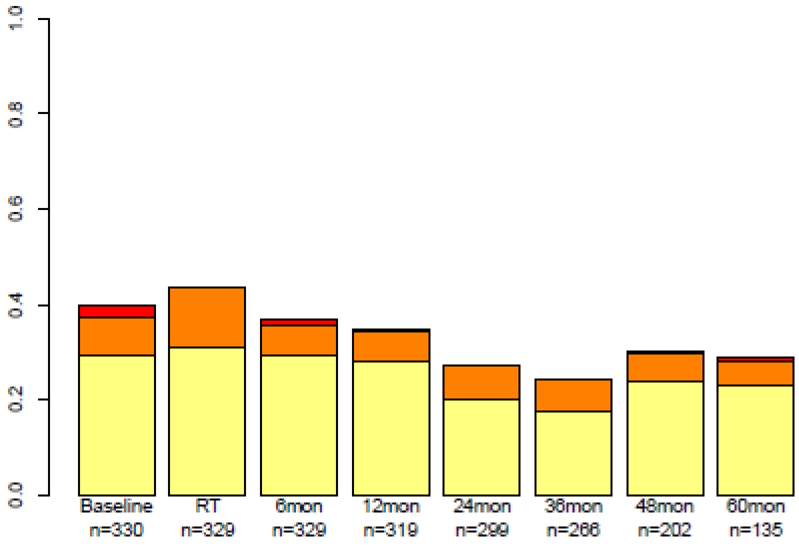

Figure S2: Incidence of the maximum physician-reported adverse events grades per patient for each timepoint at baseline, during treatment and at 6, 12, 24, 36, 48 and 60 months follow-up in the chemoradiotherapy group (A) and the radiotherapy alone group (B).

Table S1. Adverse events reported during follow-up at 12, 36 and 60 months after randomisation.
All adverse events are listed which were grade >3, or occurred in >5% of patients, or were significantly different between the study arms.

	**AE at 12 months after randomisation CTRT n=317; RT n=316**						**AE at 36 months after randomisation CTRT n=258; RT n=258**						**AE at 60 months after randomisation CTRT n=141; RT n=135**					
	**Grade 2**			**Grade 3-4**			**Grade 2**			**Grade 3-4**			**Grade 2**			**Grade 3-4**		
	**CTRT n (%)**	**RT n (%)**	**P***	**CTRT n (%)**	**RT n (%)**	**p#**	**CTRT n (%)**	**RT n (%)**	**P***	**CTRT n (%)**	**RT n (%)**	**p#**	**CTRT n (%)**	**RT n (%)**	**P***	**CTRT n (%)**	**RT n (%)**	**p#**
**Any**	103 (33)	89 (28)	**0·04**	33 (10)	21 (7)	0·09	64 (24)	48 (18)	**0·03**	22 (8)	16 (6)	0.31	43 (30)	30 (22)	**0·03**	14 (10)	8 (6)	0.27
**Any grade 3**	na	na		27 (9)	19 (6)		na	na		21 (8)	16 (6)		na	na		13 (9)	8 (6)	
**Any grade 4**	na	na		6 (2)	2 (1)		na	na		1 (0)	0 (0)		na	na		1 (1)	0 (0)	
Auditory/hearing	10 (3)	2 (1)	**0·02**	0 (0)	0 (0)	1·00	1 (0)	1 (0)	1·00	1 (0)	1 (0)	1.00	4 (3)	1 (1)	0.28	2 (1)	1 (0)	1.00
Fatigue	5 (2)	4 (1)	1·00	0 (0)	2 (1)	0·50	1 (0)	0 (0)	0·50	0 (0)	0 (0)	1.00	0 (0)	3 (2)	0.11	0 (0)	0 (0)	1.00
Hypertension	14 (4)	17 (5)	0·62	4 (1)	5 (2)	1·00	15 (6)	17 (6)	0·75	5 (2)	6 (2)	1.00	10 (7)	15 (6)	0.26	3 (2)	4 (3)	0.72
Lymphatics (edema)	8 (3)	3 (1)	0·11	2 (1)	1 (0)	0·62	3 (1)	1 (0)	0·12	2 (1)	0 (0)	0.25	3 (2)	0 (0)	0.25	0 (0)	0 (0)	1.00
**GI - any**	21 (7)	19 (6)	0·24	7 (2)	2 (1)	0·11	11 (4)	17 (6)	0·46	2 (1)	1 (0)	0.62	14 (10)	8 (6)	0.15	2 (1)	1 (1)	1.00
GI – diarrhea	11 (3)	8 (3)	0.39	1 (0)	1 (0)	1·00	4 (2)	8 (3)	0.42	1 (0)	1 (0)	1·00	6 (4)	6 (4)	1.00	0 (0)	0 (0)	1.00
GI – Ileus/ obstruction	2 (1)	3 (1)	0.77	4 (1)	2 (1)	0·45	0 (0)	0 (0)	0.49	1 (0)	0 (0)	0.50	2 (1)	1 (1)	0.37	2 (1)	0 (0)	0.50
**Hematological - any**	26 (8)	21 (7)	0·89	4 (1)	7 (2)	0·54	3 (1)	2 (1)	1·00	1 (0)	2 (1)	1.00	4 (3)	5 (4)	0.77	0 (0)	0 (0)	1.00
- Lymphocytes	24 (8)	22 (7)	0·89	4 (1)	5 (2)	1·00	3 (1)	2 (1)	1·00	1 (0)	2 (1)	1.00	3 (2)	4 (3)	0.74	0 (0)	0 (0)	1.00
**Neuropathy - any**	26 (8)	1 (0)	**<0·001**	4 (1)	1 (0)	0·22	17 (7)	2 (1)	**<0·001**	3 (1)	0 (0)	0.12	12 (8)	0 (0)	**<0·001**	1 (1)	0 (0)	1.00
Neuropathy - motor	1 (0)	0 (0)	0·22	3 (1)	1 (0)	0·37	3 (1)	2 (1)	0·45	1 (0)	0 (0)	0.50	1 (1)	0 (0)	0.50	1 (1)	0 (0)	1.00
Neuropathy - sensory	26 (8)	1 (0)	**<0·001**	4 (1)	1 (0)	0·22	17 (7)	1 (0)	**<0·001**	3 (1)	0 (0)	0.12	11 (8)	0 (0)	**<0·001**	1 (1)	0 (0)	1.00
**Pain - any**	26 (8)	22 (7)	0·18	8 (3)	3 (1)	0·14	17 (7)	15 (6)	0·24	4 (2)	0 (0)	0.06	11 (8)	5 (4)	0.27	3 (2)	3 (2)	1.00
- Joint pain	4 (1)	3 (1)	0·72	0 (0)	0 (0)	1·00	2 (1)	5 (2)	0·72	1 (0)	0 (0)	0.50	5 (4)	2 (1)	0.34	2 (1)	1 (1)	1.00
- Muscle pain	4 (1)	1 (0)	0.22	0 (0)	0 (0)	1·00	0 (0)	3 (1)	0.25	0 (0)	0 (0)	1.00	1 (1)	1 (1)	0.61	0 (0)	1 (1)	0.50
- Back/pelvic/limbs	11 (3)	5 (2)	**0·05**	3 (1)	1 (0)	0·37	4 (2)	3 (1)	0·50	1 (0)	0 (0)	0.50	0 (0)	2 (1)	0.11	0 (0)	1 (1)	0.50
- abdomen / cramps	2 (1)	5 (2)	0.77	3 (1)	2 (1)	0·69	4 (2)	1 (0)	0.12	1 (0)	0 (0)	0.50	2 (1)	0 (0)	0.12	2 (1)	0 (0)	0.50
Pulmonary - Dyspnea	2 (1)	2 (1)	1.00	0 (0)	1 (0)	1·00	1 (0)	0 (0)	1.00	0 (0)	1 (0)	1·00	2 (1)	0 (0)	0.25	0 (0)	0 (0)	1.00
GU - incontinence	8 (3)	9 (3)	1·00	1 (0)	1 (0)	1·00	8 (3)	3 (1)	0·09	1 (0)	0 (0)	0.50	7 (5)	7 (5)	1.00	0 (0)	0 (0)	1.00
GU – obstruction	0 (0)	0 (0)	1.00	0 (0)	1 (0)	1·00	0 (0)	0 (0)	1.00	0 (0)	1 (0)	1.00	0 (0)	0 (0)	1.00	0 (0)	0 (0)	1.00
GU - urinary frequency	4 (1)	8 (3)	0·26	0 (0)	1 (0)	1·00	7 (3)	4 (2)	0·38	0 (0)	0 (0)	1.00	8 (6)	1 (1)	**0.04**	0 (0)	0 (0)	1.00

Abbreviations: CTRT, combined chemotherapy and radiotherapy; RT, radiotherapy; GI, gastro-intestinal; GU, genito-urinary; NP, neutropenia; na, not applicable
AE were calculated at each time point. Per AE, the maximum grade per patient was calculated (worst ever by patient). AE grades according to CTCAE v3.0, Common Terminology Criteria for Adverse Events version 3.0
p* = significance level for grade 2, 3 and 4; p# = significance level for grade 3 and 4.
